# Oxidative stress contributes to the tamoxifen-induced killing of breast cancer cells: implications for tamoxifen therapy and resistance

**DOI:** 10.1038/srep21164

**Published:** 2016-02-17

**Authors:** Raie T. Bekele, Ganesh Venkatraman, Rong-Zong Liu, Xiaoyun Tang, Si Mi, Matthew G. K. Benesch, John R. Mackey, Roseline Godbout, Jonathan M. Curtis, Todd P. W. McMullen, David N. Brindley

**Affiliations:** 1 Signal Transduction Research Group, Department of Biochemistry, University of Alberta, Edmonton, Alberta, T6G 2S2, Canada; 2 Department of Oncology, Cross Cancer Institute, University of Alberta, Edmonton, Alberta, T6G 1Z2, Canada; 3 Department of Agricultural, Food and Nutritional Science (Lipid Chemistry Group), University of Alberta, Edmonton, Alberta, T6G 2P5, Canada; 4 Department of Surgery, Walter C Mackenzie Health Science Centre, University of Alberta, Edmonton, T6G 2R7, Alberta, Canada

## Abstract

Tamoxifen is the accepted therapy for patients with estrogen receptor-α (ERα)-positive breast cancer. However, clinical resistance to tamoxifen, as demonstrated by recurrence or progression on therapy, is frequent and precedes death from metastases. To improve breast cancer treatment it is vital to understand the mechanisms that result in tamoxifen resistance. This study shows that concentrations of tamoxifen and its metabolites, which accumulate in tumors of patients, killed both ERα-positive and ERα-negative breast cancer cells. This depended on oxidative damage and anti-oxidants rescued the cancer cells from tamoxifen-induced apoptosis. Breast cancer cells responded to tamoxifen-induced oxidation by increasing Nrf2 expression and subsequent activation of the anti-oxidant response element (ARE). This increased the transcription of anti-oxidant genes and multidrug resistance transporters. As a result, breast cancer cells are able to destroy or export toxic oxidation products leading to increased survival from tamoxifen-induced oxidative damage. These responses in cancer cells also occur in breast tumors of tamoxifen-treated mice. Additionally, high levels of expression of Nrf2, ABCC1, ABCC3 plus NAD(P)H dehydrogenase quinone-1 in breast tumors of patients at the time of diagnosis were prognostic of poor survival after tamoxifen therapy. Therefore, overcoming tamoxifen-induced activation of the ARE could increase the efficacy of tamoxifen in treating breast cancer.

Breast cancer is the most common malignancy among women in western societies^1^. After being diagnosed, primary breast tumors are surgically resected and those patients with estrogen receptor-α (ERα)-positive tumors are typically prescribed tamoxifen as an adjuvant treatment^2^,^3^,^4^. Tamoxifen is composed of triphenylethylene backbone structure and works by blocking the actions of ERα^4^. Αbout 75% of all breast tumors are ERα positive and thus tamoxifen is the most widely used therapy for breast cancer leading to tumor stabilization in about 50% of previously untreated patients with metastatic breast cancer^3^,^5^. Tamoxifen has been credited with much of the decrease in breast cancer mortality over the last decade. Nevertheless, nearly one-third of patients receiving adjuvant tamoxifen eventually experience disease relapse and almost all patients with metastatic tumors treated with tamoxifen will have progression and die from their disease^3^,^6^. The purpose of the present study was to elucidate the mechanism that leads to a decreased responsiveness to tamoxifen therapy.

Binding of the primary human estrogen, 17β-estradiol, to ERα seals the hydrophobic pocket by the helix-12 domain^7^,^8^. ERα then translocates to the nucleus, where it activates the estrogen response element and drives the transcription of estrogen-dependent genes^9^. Tamoxifen is metabolized to 4-hydroxytamoxifen (4HT)^10^,^11^, which also binds to ERα. However, this leads to a different conformational change as compared to 17β-estradiol, and the hydrophobic pocket is not sealed by helix-12 8. Consequently, 4HT blocks ERα activation.

The second nuclear estrogen receptor, ERβ, also binds tamoxifen and its metabolites with similar affinities^12^. However, the distinctions between ERα and ERβ lie in the relative levels of expression in different tissues as well as differential transcriptional responses. These differences cause ERβ to have opposite effects on proliferation, apoptosis and migration with some reports suggesting that ERβ activation is antagonistic to ERα^13^,^14^. The third estrogen receptor is a G-protein-coupled receptor, GPR30, which is predominantly localized in the endoplasmic reticulum. Tamoxifen is an agonist for GPR30 rather than an antagonist. Thus, estrogens have effects at the genomic level through ERα and ERβ but estrogens can also exert non-genomic effect through these same receptors such as mobilization of intracellular calcium^15^, activation of PI3K^16^ and adenylate cyclase^17^. Nevertheless, some of these rapid non-genomic actions could also be explained by the action of GPR30^18^.

Despite the effects of tamoxifen on estrogen-induced signaling, tamoxifen also has some beneficial effects for tumors that have low levels of ERα^2^,^19^,^20^,^21^ and tamoxifen has killing effects that are independent of ERα expression^22^,^23^,^24^,^25^,^26^,^27^,^28^,^29^,^30^. These effects have been attributed to inhibition of protein kinase C (PKC) through oxidative stress mechanisms^24^,^25^, modulation of transforming growth factor-β expression^26^ and induction of c-myc expression^27^. Furthermore, tamoxifen treatment also demonstrated some clinical activity in patients with metastatic melanomas^31^ and glioblastomas^32^. It is, therefore, evident that the therapeutic effects of tamoxifen cannot be explained entirely by its blockade of ERα or other estrogen receptors, especially since much of the earlier clinical data was established before the identification of ERβ and GPR30. As about 50% of primary breast cancers co-express both ERα and ERβ and about 15% of those tumors expressed either ERα or ERβ. Tamoxifen had more favorable outcomes in those patients with ERβ expression^33^.

Our present work was designed to improve our understanding of how tamoxifen increases the death of cancer cells and how resistance to its therapeutic actions can occur. We found that tamoxifen at concentrations, which accumulate in tumors during therapy, increases oxidative stress in ERα-positive and ERα-negative breast cancer cells, resulting in cell death. Tamoxifen-induced oxidative stress increased the accumulation of the transcription factor, Nuclear factor-erythroid 2-related factor-2 (Nrf2), which activates the anti-oxidant response element (ARE)^34^,^35^ and this contributes to chemo-resistance^36^,^37^,^38^. Tamoxifen-induced activation of the ARE in cancer cells increased the expression of anti-oxidant proteins and multi-drug resistance transporters (MDRTs). These effects in cultured breast cancer cells were also observed in breast tumors from mice treated with tamoxifen. Activation of the ARE is a defense mechanism that is designed to protect cells against oxidative damage and this inadvertently attenuates one the therapeutic effects of tamoxifen and decreases its efficacy. In support of this hypothesis, we showed that breast cancer patients, who express high levels of Nrf2 and its downstream targets NQO1, ABCC1 and ABCC3 at the time of initial diagnosis, had poorer survival following tamoxifen therapy. Hence, these results demonstrate that increased levels of Nrf2 and ARE activation could provide prognostic markers for tamoxifen-treated patients. Thus, decreasing ARE activation might serve as a strategy for improving the efficacy of tamoxifen therapy.

## Results

### Tamoxifen decreases the proliferation of ERα-positive and ERα-negative breast cancer cells

To study effects of tamoxifen, we used human MCF-7 breast cancer cells that expressed ERα and GPR30 as demonstrated by qRT-PCR and Western blotting (Supplementary Fig. 1A and 1B). We also used human MDA-MB-231 and mouse 4T1 breast cancer cells that expressed relatively little ERα and GPR30.

**Figure 1:**
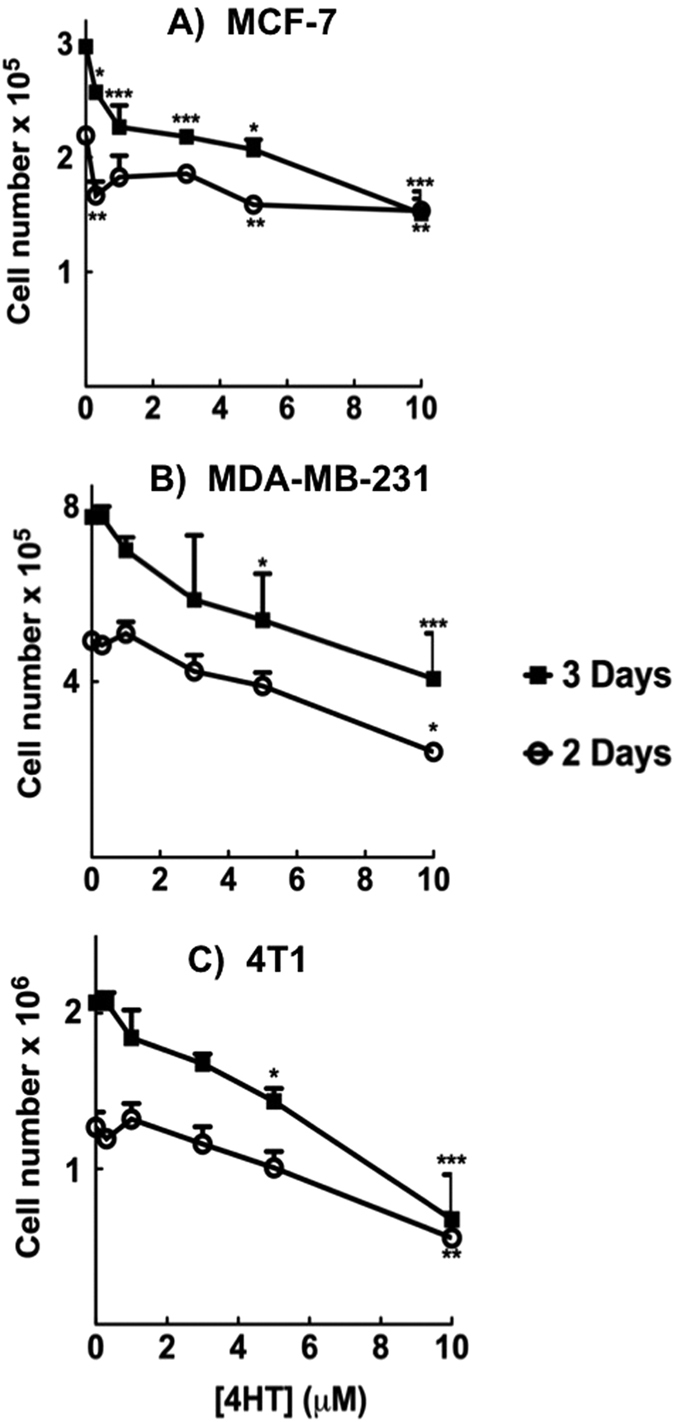
Tamoxifen slows the proliferation of breast cancer cells independently of ERα expression. (**A**) MCF-7, (**B**) MDA-MB 231 and (**C**) 4T1 breast cancer lines were treated with 4HT and the proliferation of the cells was monitored 2 and 3 days post treatment using an automated Cell Counter. Results are means ± SEM for n = 3. Significant differences were indicated with *p < 0.05, **p < 0.01 and ***p < 0.001.

Early stage breast cancer patients typically receive a 20 mg tamoxifen tablet daily for over 5 years^39^. Due to the prolonged half-life of tamoxifen and its metabolites 4-hydroxytamoxifen (4HT), N-desmethyltamoxifen (NDMT)^40^,^41^, the accumulation of tamoxifen and its metabolites in tumor tissue can easily build up to >5 μM^39^,^41^. Thus, we tested concentrations from 0.3–10 μM 4HT and measured the proliferation of ERα-positive and ERα-negative cells over three days in the presence of 10% fetal bovine serum (Fig. 1A–C). ERα-positive MCF-7 cells exhibited growth-retardation at <5 μM 4HT (Fig. 1A). ERα-negative MDA-MB-231 and 4T1 cells, required higher 4HT concentrations to inhibit proliferation (Fig. 1B,C and supplementary Fig. 1C). Nevertheless, 5–10 μM 4HT significantly blocked the proliferation of all of the cancer cells, regardless of ERα status.

**Figure 2:**
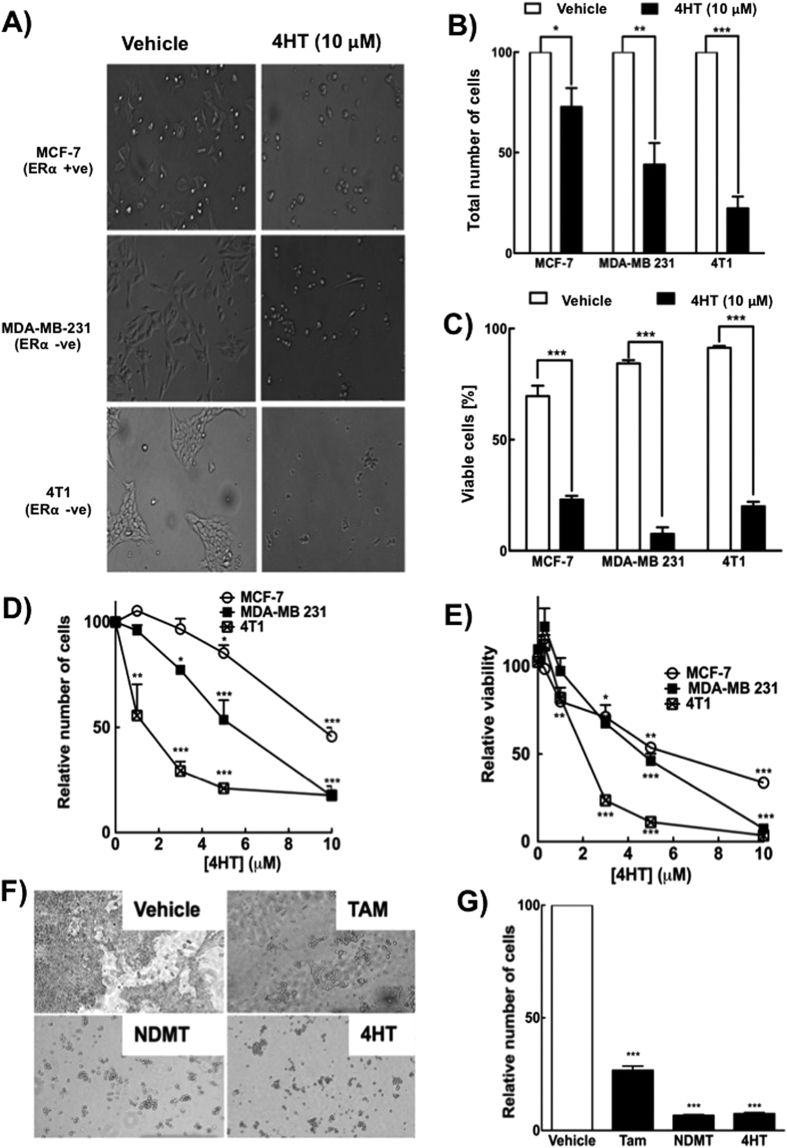
Tamoxifen and its metabolites kill both ERα positive and negative breast cancer cells. (**A**) Microscopy images of different breast cancer cells treated with or with out 10 μM 4HT for 48 h in starvation media. (**B**) Total number of cells and (**C**) percent of viable cells were quantified from microscopy images. (**D**,**E**) Breast cancer cells were treated with 0–10 μM 4HT followed by either (**D**) crystal violet staining or (**E**) MTT assay to measure the relative number of cells and the relative viability respectively. (**F**,**G**) Tamoxifen and its metabolites NDMT and 4HT induced 4T1 cell killing as shown in the (**F**) microscopy images and (**G**) crystal violet staining. Results are means ± SEM for n = 3. Significant differences were indicated with *p < 0.05, **p < 0.01 and ***p < 0.001.

To study this phenomenon further we used serum-free medium, which was devoid of growth factors including estrogen. Different cancer cell lines were treated with 10 μM 4HT for 48 h, which resulted in cell death for both ERα-negative and ERα-positive cells (Fig. 2A). 4HT treatment decreased the number of cells remaining on the dish (Fig. 2B) and the number of viable cells (Fig. 2C). Crystal violet staining and the MTT assay were also used to determine cell numbers (Fig. 2D) and metabolic activity of the cells (Fig. 2E). 4T1 cells were killed by 1 μM 4HT, whereas MDA-MB-231 and MCF-7 cells responded from >3 μM 4HT (Fig. 2D,E).

Overall, results from Figs 1 and 2 show that tamoxifen and its metabolites (Fig. 2F,G) kill both ERα-positive and ERα-negative cells at clinically relevant concentrations. We, therefore, determined the mechanisms behind these actions of tamoxifen and its metabolites.

### Tamoxifen treatment induces oxidative stress

**Figure 3:**
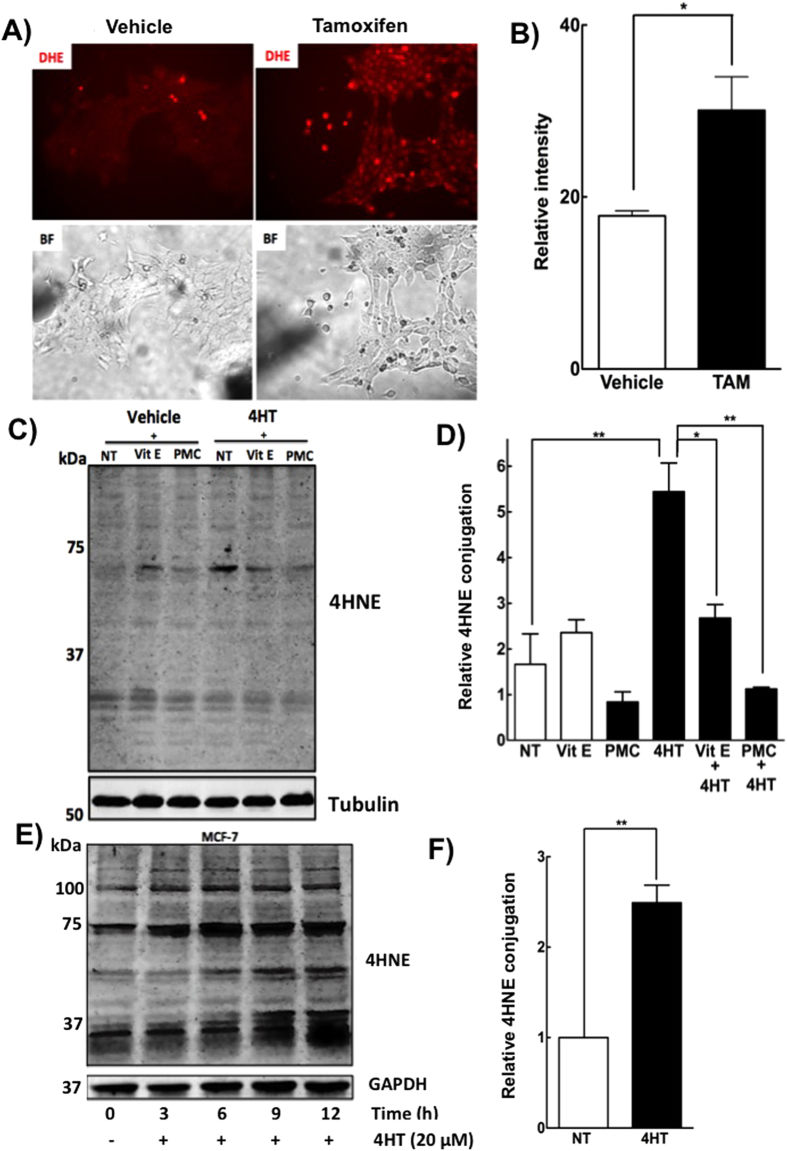
Tamoxifen treatment induces oxidative stress. (**A**) Representative fluorescence microscopy images from DHE stained 4T1 cells followed by either vehicle or 10 μM tamoxifen treatment. (**B**) Quantification of relative superoxide formation as measured by DHE staining intensity. (**C**) 4T1 cells were pretreated with the antioxidants vitamin E (100 μM) or PMC (100 μM) for 3 h followed by 24 h treatment with 10 μM 4HT. The extent lipid peroxidation was detected by western blot using 4HNE antibody; cropped representative images are shown. (**D**) Results of the relative 4HNE conjugations were quantified from western blotting. (**E**) Western blots showing time-dependent conjugation of cell proteins with 4HNE after treatment with 4HT in MCF-7 cells, cropped representative images are shown and (**F**) the corresponding quantification of this 4HNE conjugation at the 12 h time point. Results are means ± SEM for n = 3. Significant differences were indicated with *p < 0.05 and **p < 0.01.

Tamoxifen is hydrophobic and it accumulates rapidly in phospholipid bilayers of membranes where it is postulated to induce oxidative stress^24^. To investigate this, we pretreated 4T1 breast cancer cells with the superoxide indicator, dihydroethidium (DHE), which is oxidized to produce a bright fluorescent red color when it interacts with superoxide. Treatment with 10 μM tamoxifen for 24 h significantly increased the red staining in 4T1 cells, indicating a higher level of superoxide generation (Fig. 3A,B). Since this could lead to lipid peroxidation, we measured the production of 4-hydroxynonenal (4HNE), which results from the oxidation of membrane lipids. 4HNE conjugates with cell proteins and this effect can be visualized by Western blotting with an anti-4HNE antibody^42^. Treatment of 4T1 cells with 10 μM 4HT for 24 h significantly increased the conjugation of proteins at 65 kDa with 4HNE and this effect was partially blocked with the antioxidants, vitamin E and/or PMC (2,2,5,7,8-pentamethyl-6-chromanol, a vitamin E moiety without a lipid tail) (Fig. 3C,D). Increased conjugation of cell proteins with 4HNE was also observed in MCF-7 cells treated for 3–12 h with 20 μM 4HT (Fig. 3E,F). Hence, treatment of breast cancer cells with 4HT increased in the oxidation of membrane lipids (Fig. 3 and Supplementary Fig. 2).

### Tamoxifen increases the levels of ceramide, JNK phosphorylation and markers of apoptosis

**Figure 4:**
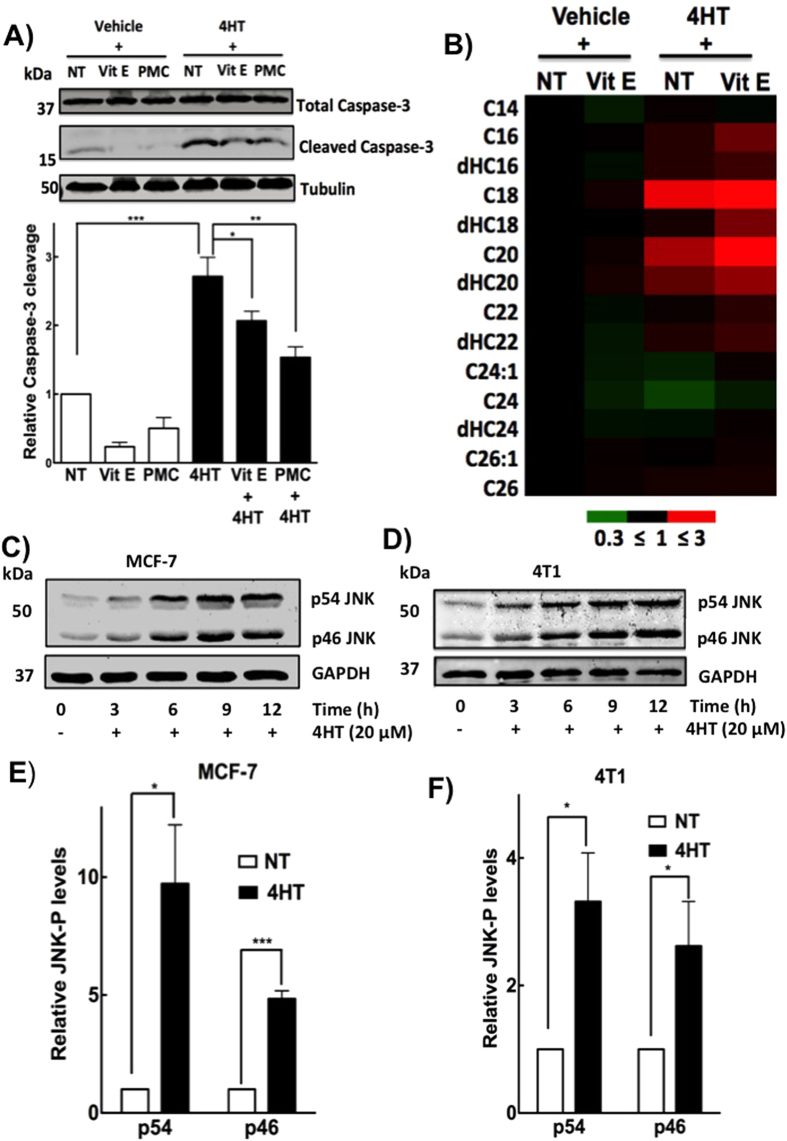
Tamoxifen induces apoptosis, stimulates the accumulation of different ceramides species and increases phosphorylated JNK. (**A**) Treatment of 4T1 cells with 10 μM 4HT leads to cleavage of caspase-3 and this is partially rescued with vitamin E (100 μM) or its analog PMC (100 μM), shown by the cropped representative blots (top). The respective quantification is shown from n = 3 experiments (bottom). (**B**) Relative levels of different ceramide species in 4T1 cells that were treated with vehicle, 100 μM vitamin E, 10 μM 4HT or the combined vitamin E and 4HT. Results were from n = 4 measurements and are depicted on a heat map with black representing no change, green showing a decrease and red showing a significant increase. (**C**–**F**) Western-blot for phosphorylated p54 JNK and p46 JNK after 3, 6, 9 and 12 h of 4HT treatment in (**C**) MCF-7 cells and (**D**) 4T1 cells, representative cropped blots are shown. Corresponding quantification for the phosphorylated p54 JNK and p46 JNK at 12-h mark in (**E**) MCF-7 and (**F**) 4T1 cells from n = 3 experiments. Significant differences were indicated with *p < 0.05, **p < 0.01 and ***p < 0.001.

We next determined the effects of tamoxifen on caspase-3 activation, which is known to cleave poly (ADP-ribose) polymerase (PARP) and other proteins leading to apoptosis. Caspase-3 activation, which occurs through proteolytic cleavage resulting in an increase in a 17-kDa fragment, is indicative of apoptosis^43^. Treatment of 4T1 cells for 24 h with 10 μM 4HT increased caspase-3 cleavage and this increase was partially blocked by the anti-oxidants, vitamin E or PMC (Fig. 4A). Treatment of MCF-7, MDA-MB-231 and 4T1 cells with 4HT also increased PARP cleavage (Supplementary Fig. 3A,B). However, vitamin E was unable to rescue cells from 4HT-induced PARP cleavage (Supplementary Fig. 3C) indicating some caspase-3-independent cleavage of PARP^44^.

Cell death following radiotherapy and chemotherapeutic agents is often accompanied by the accumulation of ceramides. These are lipid-signaling mediators that are highly responsive to stress and relay apoptotic messages leading to activation of caspases^45^,^46^,^47^. Since tamoxifen induces oxidative stress and leads to apoptosis, we investigated if this was accompanied by increased ceramide accumulation. Treatment of 4T1 cells with 10 μM 4HT significantly increased (p < 0.05) the accumulation of C16-, dHC16-, C18-, C20-, dHC20-, dHC22-ceramides (Fig. 4B, Supplementary Fig. 4). However, vitamin E did not block ceramide accumulation (Fig. 4B). 4HT also increased ceramide concentrations in MCF-7 and MDA-MB 231 cells (Supplementary Fig. 4).

In addition, we determined the effects of 4HT on JNK, which belongs to a family of stress-activated protein kinases. JNK activation is often linked to oxidative stress and increased ceramide levels^48^. JNK isoforms, p54 and p46, were activated through phosphorylation of Thr183/Tyr185 residues following 4HT treatment in both 4T1 and MCF-7 cells (Fig. 4C–F). However, vitamin E did not block the increases in phosphorylated JNK (Supplementary Fig. 5A) similar to the effects on ceramide accumulation.

### Tamoxifen increased Nrf2 levels and activates the anti-oxidant response element

**Figure 5:**
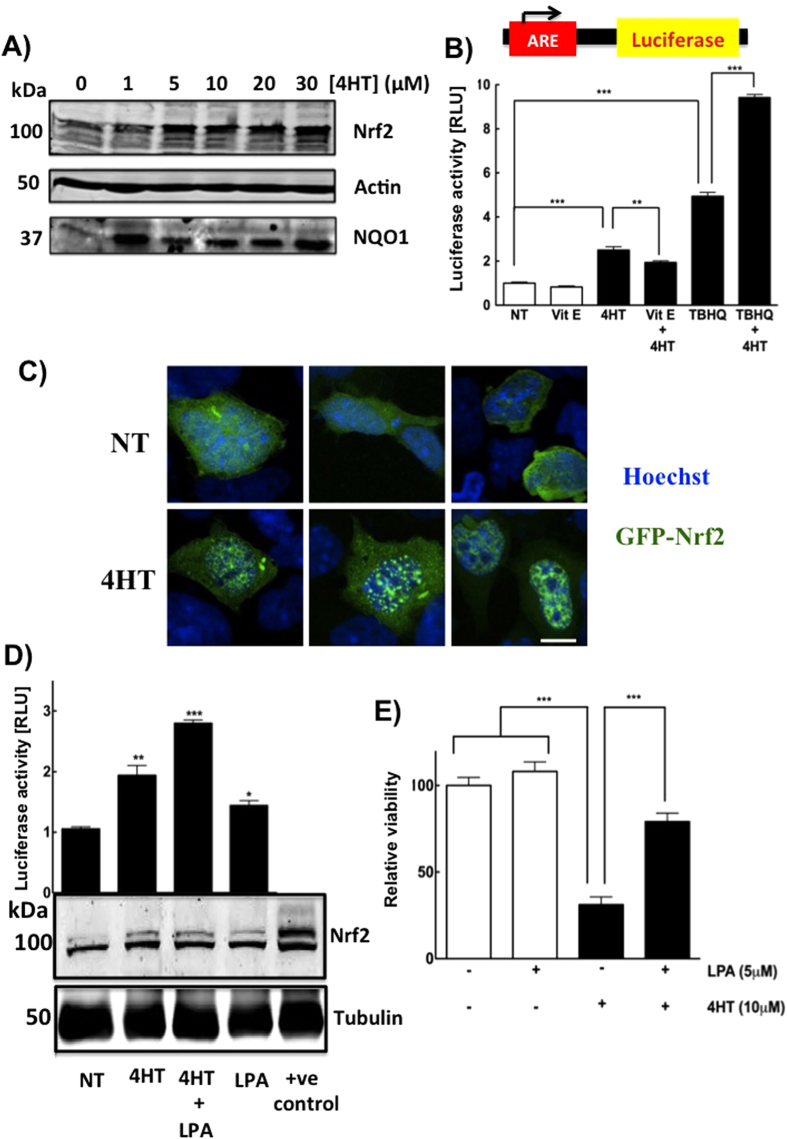
Tamoxifen treatment increases Nrf2 levels and translocates Nrf2 to the nucleus causing an increase in transcription through the ARE. (**A**) 4HT stimulates a dose-dependent increase in Nrf2 levels and the anti-oxidant gene NQO1 in MCF-7 cells, cropped representative images are shown. (**B**) Luciferase activity assay after treatment with vehicle, 100 μM vitamin E and 10 μM TBHQ alone or in combination with 10 μM 4HT in MCF-7 cells stably expressing a luciferase reporter gene downstream of the ARE. (**C**) Confocal microscopy images of HEK 293 cells transfected with GFP-Nrf2 and treated with either vehicle or with 4HT. Hoechst was used to stain for nuclei. (**D**) Luciferase activity (top) and representative cropped Western blot for Nrf2 (bottom) after treating cells with the vehicle and LPA alone or in conjunction with 10 μM 4HT. (**E**) Treatment of MCF-7 cells with 5 μM LPA rescued cells from tamoxifen-induced cell killing. Results are means ± SEM for n = 3. Significant differences were indicated with *p < 0.05, **p < 0.01 and ***p < 0.001.

Since tamoxifen treatment increases oxidative stress, we tested if it would increase accumulation of the transcription factor, Nrf2. MCF-7 cells were treated with 1 to 30 μM 4HT which increased the levels of Nrf2 and NAD(P)H dehydrogenase quinone-1 (NQO1), which is a downstream anti-oxidant gene (Fig. 5A).

To further corroborate the role of Nrf2 in response to 4HT, we used MCF-7 cells stably expressing the ARE sequence upstream of a luciferase reporter gene. 4HT increased luciferase expression and this increase was partially blocked by vitamin E. The addition of 4HT with tert-butylhydroquinone (TBHQ) (a known activator of ARE and Nrf2 stability) further increased luciferase expression (Fig. 5B). Tamoxifen treatment also increased the nuclear localization of GFP-tagged Nrf2 (Fig. 5C).

We showed previously that lysophosphatidate (LPA) by stimulating LPA_1_ receptors is a natural activator of the ARE through stabilizing of Nrf2. This leads to the subsequent transcription of anti-oxidant genes and MDRTs contributing to chemo-resistance associated with the effect of doxorubicin^49^. Hence, we hypothesized that LPA could antagonize the oxidative actions of tamoxifen by a similar mechanism. As predicted treatment of MCF-7 cells with 4HT in the presence of LPA further increased ARE-dependent luciferase expression (Fig. 5D) and also blocked the killing effects of tamoxifen (Fig. 5E).

### Effects of tamoxifen on tumor growth in mouse model of breast cancer

**Figure 6:**
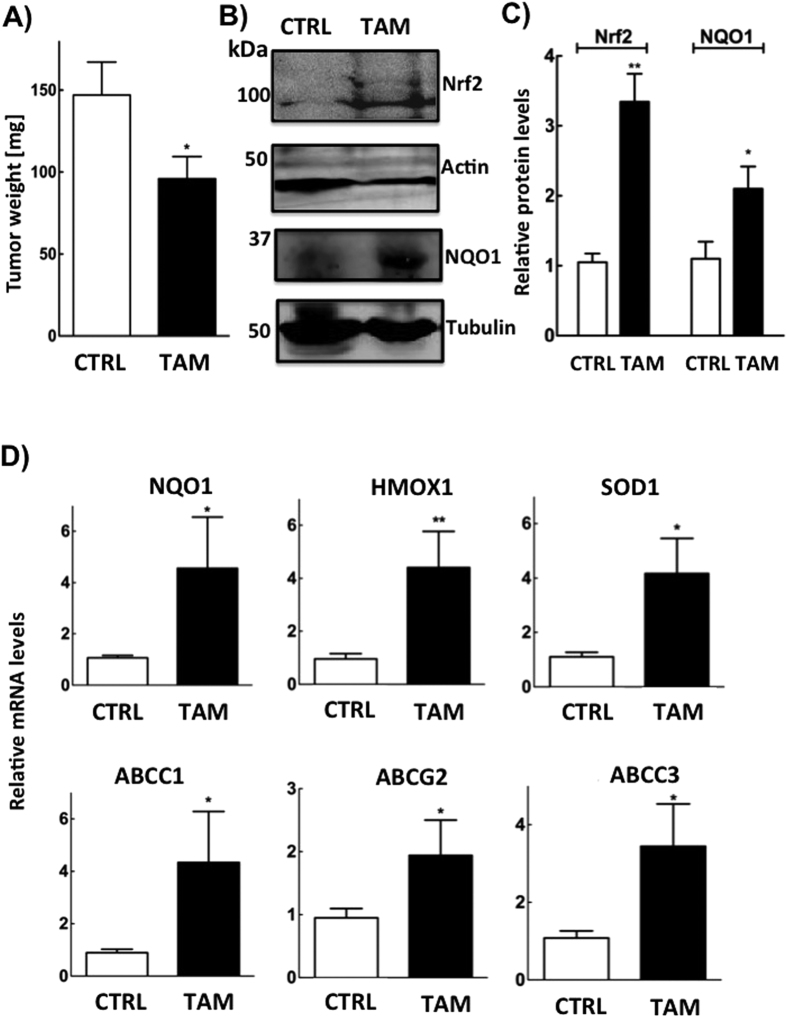
Tamoxifen decreases the tumor burden in mice and increases Nrf2 dependent genes in an orthotopic mouse model of breast cancer. (**A**) Primary tumors were excised and weighed (results from n = 6 mice per group). (**B**) Western blots showing representative cropped images for the expressions of Nrf2 and NQO1 in tumors from control and tamoxifen-treated mice with (**C**) the corresponding quantification for Nrf2 relative to actin and NQO1 relative to tubulin. (**D**) Shows the relative mRNA levels for NQO1, HMOX1, SOD1, ABCC1, ABCG2 and ABCC3 in control versus tamoxifen treated mice. Significant differences were indicated with *p < 0.05 and **p < 0.01.

We used an orthotopic syngeneic mouse model by injecting 4T1 breast cancer cells, which do not express ERα, into the mammary fat pad of female Balb/c mice to test whether the effects of tamoxifen observed in cell culture could be recapitulated *in vivo*. The tamoxifen treatment regimen (Methods Section) was well tolerated by the animals as shown by the body weight measurements (Supplementary Fig. 6). This syngeneic model produced a substantial tumor burden within 10 day and tamoxifen treatment decreased breast tumor weight significantly by about 35% compared to the control group (Fig. 6A). Tamoxifen treated mice had significantly increased the protein expression levels for Nrf2 and NQO1 in their breast tumors (Fig. 6B,C). Tamoxifen also increased mRNA expression of NQO1, heme oxygenase 1 (HMOX1), superoxide dismutase 1 (SOD1) and the MDRTs: ABCC1, ABCC3 and ABCG2 (Fig. 6D), which all belong to the ATP-binding cassette transporters (ABC) family of proteins.

### Prognostic values of Nrf2, ABCC1, ABCC3 and NQO1 in human cancer patients treated with tamoxifen

To determine the validity of our findings from the cell culture and the syngeneic mouse model to responses in human patients, we used a collection of human breast tumors included in the Breast Cancer Relapsing Early Determinants study^50^. This collection included 176 patients diagnosed with primary breast cancer who had their tumors resected before treatment (Supplementary Table 1). Of these patients, 64% had ERα-positive tumors and ~84% of these patients were treated with tamoxifen. The ERα-positive patients who were not treated with tamoxifen were mainly postmenopausal and they received aromatase inhibitors. Other treatments included trastuzumab for those with Human Epidermal growth factor Receptor 2 (HER2) positive tumors, or anthracycline for high-risk node-negative disease and adjuvant cytotoxic chemotherapy for those with high-risk features. Additional information about the clinical and pathological features of the breast cancer cases used in this study is provided in the Supplementary Table 1.

**Figure 7:**
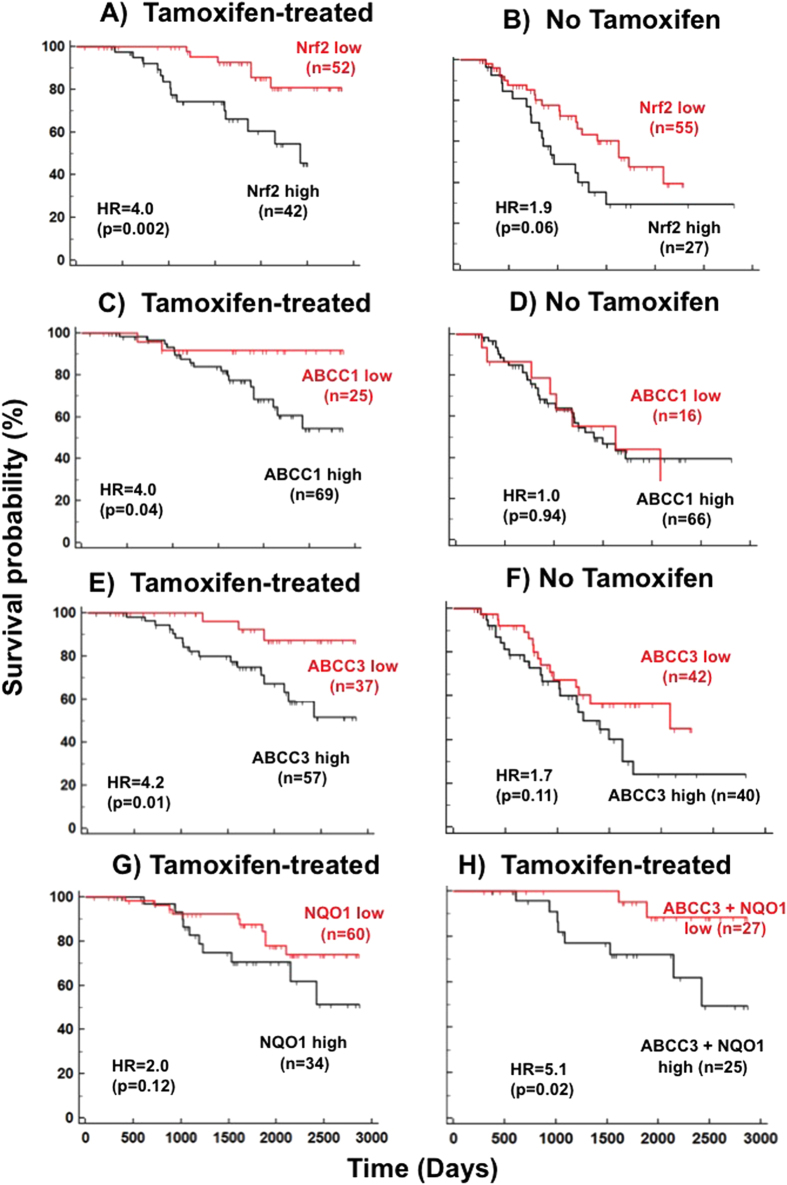
The gene expression profiles of Nrf2, ABCC1, ABCC3 and NQO1 serve as a good prognostic marker for the survival of human breast cancer patients treated with tamoxifen. Patients stratified as high and low expressers of (**A**,**B**) Nrf2, (**C**,**D**) ABCC1, (**E**,**F**) ABCC3, (**G**) NQO1 and (**H**) NQO1 and ABCC3 together. The survival data were plotted from patients who received tamoxifen treatment (**A,C**,**E**,**G**,**H**) or patients with NO tamoxifen treatment (**B**,**D**,**F**).

Gene expression profiles from the resected tumors were determined and a prognostic analysis for the survival probability of the patients was performed for tamoxifen-treated patients and those that did not receive tamoxifen. Patients with tumors that had high expression of Nrf2 had a significantly lower survival probability of p = 0.002 and a hazard ratio (HR) value of 4.0 for the tamoxifen-treated cohort. This indicated a 4-fold decrease in overall survival probability as compared with the low Nrf2 expressing group (Fig. 7A). However, in patients that did not receive tamoxifen, the difference in survival probability was not statistically significant (p = 0.06) but there was a trend towards better survival in the low Nrf2 expresser group with HR value of 1.9 (Fig. 7B).

For ABCC1 and ABCC3 the overall survival probability within the tamoxifen-treated group showed a better survival probability for the low ABCC1 expressers (p = 0.04) and HR value of 4.0 (Fig. 7C) and p = 0.01 and an HR value of 4.2 for ABCC3 (Fig. 7E). Nevertheless in the patients without tamoxifen treatment, the results were not statistically significant and did not show a trend for ABCC1 (p = 0.94 and HR value of 1.0) (Fig. 7D). However, there was a trend towards better survival in the low ABCC3 expresser group with HR value of 1.7 (Fig. 7F).

For NQO1 within the tamoxifen-treated patients there was only a trend towards increased survival (HR = 2) in the low NQO1 expressers (Fig. 7G) and patients that were not treated with tamoxifen did not show any prognostic value (Supplementary Fig. 7A). However, we did identify a statistically significant (p < 0.0001) correlation between the expressions of NQO1 and ABCC3 (Supplementary Fig. 7B). This prompted us to analyze the survival probability by grouping patients into double high expressers for NQO1 and ABCC3 and compare them to double low expressers. The double high expressers had a significantly lower survival with p = 0.02 and an HR value of 5.1 with in the tamoxifen-treated group (Fig. 7H).

## Discussion

The present work expands our understanding of how tamoxifen kills breast cancer cells and it elucidates the adaptive mechanisms that decrease the efficacy of tamoxifen treatment. We showed that concentrations of tamoxifen and its metabolites (≥5 μM), which occur in breast tumors of patients^39^,^41^, kill breast cancer cells independently of ERα expression. A major part of this effect was through oxidative stress caused by tamoxifen accumulation since the cancer cells were partially protected from apoptosis by the anti-oxidants, vitamin E and PMC.

Tamoxifen partitions into lipid membranes resulting in increased oxidative damage^24^. We demonstrated this since tamoxifen treatment increased the formation of superoxides and the lipid peroxidation product 4HNE in breast cancer cells. Other studies showed that adding tamoxifen to calf thymus DNA in the presence of microsomal preparations increased 8-hydroxy-2′-deoxyguanosine levels (a marker for oxidative stress) on the DNA. This oxidative damage on DNA was diminished by adding SOD1^51^. Tamoxifen also inactivates protein kinase C through oxidative stress and this effect was reversed in the presence of SOD1 or vitamin E^24^. Also, studies using MCF-7 derived xenografts tumors in athymic mice showed that tamoxifen treatment increased SOD1 levels and long-term treatment of these tumors with tamoxifen led to tamoxifen-resistance^52^. We showed that blocking this oxidative stress partially rescued breast cancer cells from lipid peroxidation and subsequent apoptosis. This tamoxifen-induced generation of reactive oxygen species (ROS) could be mediated by the membrane bound enzyme, NADPH oxidase, since studies showed its inhibition blocked tamoxifen induced ROS production and apoptosis in human hepatoblastoma cells^53^. Hence, oxidative stress contributes to killing cancer cells during tamoxifen therapy, but this action also has the consequence of increasing tamoxifen-resistance.

We substantiated this conclusion by showing that treatment of cancer cells with 4HT increased Nrf2 stability and gene transcription through the ARE. This effect was also observed in our mouse model of breast cancer, using ERα-negative 4T1 cells. Tamoxifen treatment decreased tumor size and this was accompanied by increased Nrf2 expression in the breast tumors. The latter result explained the tamoxifen-induced increases in the expressions of NQO1, HMOX1, SOD1, ABCC1, ABCG2 and ABCC3, which are transcribed downstream of the ARE. These results are also compatible with the characteristics of MCF-7 breast cancer cells, which were selected for tamoxifen-resistance. Resistant cells had increased expression of Nrf2 and knocking down Nrf2 expression decreased the high expression of anti-oxidant genes in the tamoxifen resistant cells^54^. Knockdown of Nrf2, in the same study, also increased tamoxifen-induced cell death and the increased expression of the anti-oxidant genes did not depend on ERα signaling. We conclude that the oxidative damage caused by tamoxifen elicits an anti-oxidant response, which attempts to protect the cells from cell death.

Our studies showed that <5 μM tamoxifen retarded the growth of ERα-positive MCF-7 cells, but these concentrations had little effect in ERα-negative MDA-MB-231 and 4T1 cells. Higher tamoxifen concentrations (>5 μM) killed breast cancer cells independently of ERα expression. This ERα independent killing was also observed in studies with ERα negative BT-20 breast cancer cells^30^, ovarian A2780 cancer cells, T-leukemic Jurkat cells^55^ and hepatoblastoma cells^53^. This concentration-dependent action of tamoxifen is especially significant since tamoxifen and its metabolites can accumulate preferentially to >5 μM in breast tumors compared to normal breast tissue^39^,^41^. At these concentrations, tamoxifen has a therapeutic action through oxidative damage that is independent of ERα. However, cancer cells then mount an anti-oxidant response that decreases the efficacy of the oxidative component of tamoxifen therapy. This cytotoxic accumulation of tamoxifen could be aided by Antiestrogen Binding Site (AEBS) to which tamoxifen has high affinity as opposed to estrogen. AEBS are found in various tissues including estrogen receptor-negative breast cancer cells^56^.

We also found that tamoxifen stimulated the accumulation of several ceramide species, increased PARP cleavage and JNK activation. However, these effects were not blocked by vitamin E. This indicates that tamoxifen also initiates mechanisms of cell death that are independent of oxidative stress. Nevertheless previous studies have linked oxidative stress to the formation of ceramide and subsequent JNK activation^45^. We were also able to activate JNK using C2-ceramide (Supplementary Fig. 5B). Moreover, tamoxifen-induced JNK activation was not reversed by inhibiting ASK1 (Apoptosis signal regulating kinase, Supplementary Fig. 5B), which was linked in other studies to JNK activation^57^. Also, JNK inhibition did not block caspase-3 activation. Interestingly, several studies showed that cytokines and stress stimuli such as TNF-α, interleukins, FAS ligand, heat shock, UV irradiation and also stressors that deplete glutathione such as ROS and 4HNE can activate sphingomyelinase, which leads to increased ceramide production^58^,^59^,^60^. Moreover, ceramide formation activates caspase-8-dependent, but caspase-3-independent, necrosis in lymphoid cells treated with FAS ligand^46^. If ceramide formation induces caspase-3-independent cell killing, it would explain why vitamin E could block caspase-3 activity, but not block ceramide accumulation and PARP cleavage. Other studies also showed caspase-3-independent PARP cleavage^44^ and this is also evident from our work on PARP cleavage in MCF-7 cells (Supplementary Fig. 3), which are caspase-3 deficient^61^.

Our work with the syngeneic mouse model of breast cancer using 4T1 cells demonstrates that tamoxifen decreased tumor growth in an ERα-negative breast cancer. This was accompanied by increased expression of Nrf2, antioxidant genes (NQO1, HMOX1, SOD1) and the multidrug resistant transporters (ABCC1, ABCG2, ABCC3). These results confirm that tamoxifen induces oxidative damage in breast tumors *in vivo* and supports our hypothesis that ARE activation could contribute to the development of tamoxifen resistance. Other studies also showed that tamoxifen attenuates tumor growth in a xenograft model with ERα-negative MDA-MB-468 human breast cancer cells. The authors attributed this to the degradation of cancerous inhibitor of protein phosphatase-2A (PP2A) (CIP2A) by tamoxifen^62^. CIP2A inhibits PP2A, whereas 4HNE activates PP2A^63^. This raises the possibility that tamoxifen-induced degradation of CIP2A occurs through increased 4HNE formation leading to PP2A activation. PP2A activation leads to subsequent inactivation of survival proteins such as Akt, thus contributing to apoptosis^62^.

The proposal that oxidative stress could play a role in the therapeutic effects of tamoxifen is further supported by our analysis of the survival of breast cancer patients treated with tamoxifen. Those patients, who had ERα-positive breast tumors with low expression of Nrf2, ABCC1, ABCC3 and NQO1 at the time diagnosis, had a better prognosis after tamoxifen treatment than those patients that were high expressers, with an HR value as high as 4.2. This HR value further increased to 5.2 when considering patients that were high expressers for both NQO1 and ABCC3. High Nrf2 and ABCC3 expression was also associated with poor prognosis for other treatments including trastuzumab, anthracycline and taxanes. For Nrf2 this was expected since Nrf2-induced activation of the ARE is commonly associated with chemo-resistance^36^,^37^,^38^. By contrast in our work, high ABCC1 expression was only prognostic of a poor outcome within the tamoxifen-treated group. These associations are not predicted from the classical action of tamoxifen through blocking ERα signaling. They support our conclusion that tamoxifen-induced killing of cancer cells through oxidative damage is an important component of tamoxifen action.

**Figure 8:**
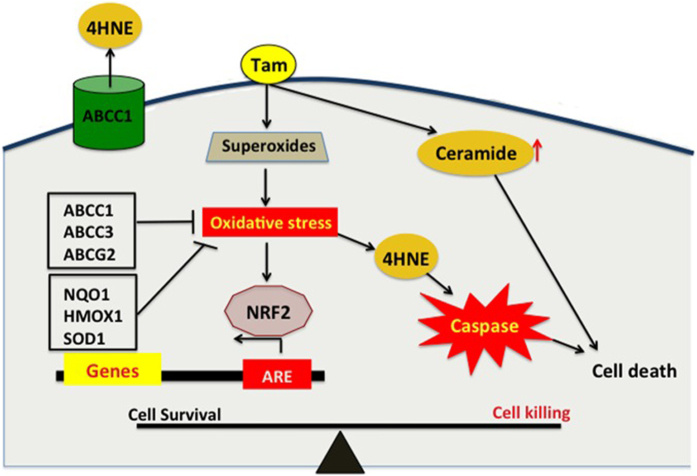
Proposed signaling scheme for the effects of tamoxifen in breast cancer and the development of resistance. Tamoxifen embeds itself in the lipid bilayer and generates superoxide, which causes a lipid peroxidation and subsequent 4HNE formation. 4HNE activates caspase-3 and leads to cell killing. In addition, tamoxifen also increases ceramide levels in cells. Breast cancer cells respond to the oxidative stress environment by increasing Nrf2 levels and thereby activating ARE leading to the expression of antioxidant genes (NQO1, HMOX1, SOD1) and the multidrug resistant transporters (ABCC1, ABCG2, ABCC3), which mitigate the effects of tamoxifen-induced oxidative stress and thus contributing to resistance.

This conclusion is compatible with the observation that ABCC1 exports the toxic oxidation product, 4HNE, from cancer cells^64^ (Fig. 8). Additionally, ABCC2 is also overexpressed in tamoxifen-resistant cells^65^. Genotyping studies looking at single nucleotide polymorphisms (SNPs) have also revealed that specific variants of the ABCC2 gene were prognostic of tumor recurrence during tamoxifen monotherapy^66^. SNPs could alter the steady state transcript level of the transporter^67^, possibly increasing ABCC levels in some patients. Other reports showed that ABCC1, ABCC11 and ABCG2 are highly over-expressed in subtypes of aggressive breast cancer and that increased expressions of ABCC1 and ABCC11 were significantly associated with shorter disease-free survival^68^. This survival study was analyzed over a short median follow up of 40 months and interpretation could be further complicated because of patients receiving neo-adjuvant therapy. Nevertheless, such studies coupled with our analysis show the importance of the ABCC family of transporters in influencing the outcome of tamoxifen therapy. Despite this, there is no clear evidence that tamoxifen is exported by ABCC1 or ABCC3^69^. Instead, glucuronide conjugates of tamoxifen could be exported through ABCC transporters^70^,^71^. On the other hand, tamoxifen is known to bind to the transporter ABCB1, but ABCB1 does not transport tamoxifen. Instead, tamoxifen binding to ABCB1 blocks its ability to transport other xenobiotics^72^.

Despite such effects of tamoxifen, there is not much clinical data showing the benefit of tamoxifen in truly negative breast tumors. The Early Breast Cancer Trialists’ Collaborative Group (EBCTCG) performed meta-analysis of 55 randomized clinical trials, which demonstrated a substantial survival advantage after tamoxifen treatment in ERαpositive breast cancer patients^2^. Thus most of the subsequent studies focused on elucidating the action of tamoxifen only in the context of ERα. Nevertheless, EBCTCG reported that tamoxifen had some activity in the patients with very low, or no ERα. A later study by Dowsett *et al.*^21^ showed that ERα-negative breast cancer patients also showed a strong trend to benefit from tamoxifen. Glioblastoma patients also benefit from high dose tamoxifen treatment, which was thought to be due to the effect of tamoxifen on PKC. Additionally, the activity of tamoxifen in advanced melanomas^31^ indicates the utility of tamoxifen in cases where ERs are not thought to be important. Nonetheless, it is often difficult to rule out the involvement of ERα and ERβ since other tissues such as gliomas also express the receptors^73^.

A previous study showed that the binding of antiestrogens to ERα and particularly to ERβ can induce the expression of the antioxidant NQO1^74^. Binding of low μM concentrations of antiestrogen to the ERs caused the receptors to complex with the ARE leading to the expression NQO1^74^. The authors of that study suggested two pathways for ARE activation and induction of NQO1, one that depended on ERs and the other that was independent of ERs^74^. Since Nrf2 is a master regulator controlling the expressing of antioxidant genes the major component bound to the ARE is most probably Nrf2 and the ERs could thus function in a cooperative role in this system. Our result with cytotoxic concentrations of tamoxifen (>5 μM) could thus activate both pathways to elicit an antioxidant response. ERs can also regulate gene expression without directly interacting with DNA by influencing other transcription factors through their binding to co-regulatory proteins, as in the case with AP-1 complex^9^. Similarly ERs could potentially be involved in the anti-oxidant gene expression involving Nrf2 through recruiting co-regulatory proteins.

Our studies indicate that it could be beneficial to decrease the expression of Nrf2, ABCC1, ABCC3 and NQO1 as an adjuvant to improve tamoxifen therapy (Fig. 8). One possibility is by blocking the activation of cancer cells by LPA. This growth factor produces resistance to the effects of paclitaxel^75^, cisplatins^76^, doxorubicin^49^ and also tamoxifen, as we showed in this work. Significantly, an important mechanism for this resistance is the role of LPA in increasing Nrf2 stability and activation of the ARE. These effects of LPA on breast tumors can be attenuated by blocking LPA production through inhibition of autotaxin^77^, or using an inhibitory antibody against LPA^78^. Proof of principle for this approach was obtained in a mouse model of breast cancer where autotaxin inhibition decreased ARE activation and the consequent expression of anti-oxidant proteins and MDRT^49^. Other strategies would involve using inhibitors that target Nrf2 such as, the quassinoid brusatol^79^, the alkaloid trigonelline^80^ and the flavonoid luteolin^81^, all of which showed efficacy in inhibiting Nrf2 activity and prevented chemo-resistance. Employing such inhibitors, as an adjuvant to tamoxifen could be beneficial, especially since knockdown of Nrf2 increased tamoxifen-induced cell death in tamoxifen resistant cells^54^.

In summary this study emphasizes the importance of the oxidative response of cancer cells to tamoxifen treatment. On the one hand, this oxidative damage to cancer cells has a positive therapeutic effect of killing the cancer cells, but on the other it amplifies the anti-oxidant response leading to increased expression of MDRT and anti-oxidant genes. This latter effect protects cancer cells from further oxidative damage and thereby produces resistance to the continued therapeutic effects of tamoxifen. Thus, evaluating breast tumors of patients for the expression of Nrf2, ABCC1, ABCC3 and NQO1 warrants formal assessment as predictive markers for tamoxifen response. Furthermore, blocking the activation of the ARE during tamoxifen therapy could improve its efficacy and alleviate acquired resistance.

## Materials and Methods

### Reagents

Ceramide standards and oleoyl-lysophosphatidate (LPA) were purchased from Avanti Polar Lipids (Alabaster, AL, USA). MTT reagent, crystal violet, 4-hydroxytamoxifen (4HT), N-desmethyltamoxifen (NDMT), vitamin E (α-Tocopherol), TBHQ (tert-Butylhydroquinone), PMC (2,2,5,7,8-Pentamethyl-6-chromanol), DMSO, protease inhibitors cocktail, sodium orthovanadate and formic acid were from Sigma (Oakville, ON, Canada). Acetic acid, acetonitrile, 2-propanol and methanol were purchased from Fisher Scientific (Ottawa, Ontario, Canada). Inhibitor for JNK (JNKi) and ASK1 (ASKi) were from Tocris Bioscience (Ellisville, MO, USA), Tamoxifen (TAM) and Microcystin-LR were from Cayman Chemical Co (Ann Arbor, MI, USA), Matrigel was from BD Biosciences (Mississauga, ON, Canada) and peanut oil was from Sobeys (Edmonton, AB, Canada). Primary antibodies were obtained as follows: Anti- ER-alpha, Anti-PARP, Anti-Cleaved Caspase-3, Anti-PARP and Anti-P-SAPK/JNK were from Cell Signaling (Danvers, MA, USA), Anti-4HNE and Anti β-actin was from Abcam (Toronto, ON, Canada), Anti-GAPDH and Anti-α-Tubulin was from (Sigma), Anti-Nrf2 (H-300) and Anti-NQO1 (A180) were from Santa Cruz (Santa Cruz, CA, USA), Anti-Calnexin was from Enzo Life Sciences (Farmingdale, NY, USA) and Anti-GPR30 was from Genscript (Piscataway, NJ, USA). Anti-rabbit and Anti-mouse secondary antibodies conjugated to infrared fluorescent dyes (IRDye) were purchased from LI-COR Biosciences (Lincoln, NE, USA). All cell lines were purchased from ATCC (Manassas, VA, USA). MCF-7 cells used in luciferase assay, stably expressed an inducible antioxidant response element upstream to a luciferase reporter gene^82^ and were the kind gift of Prof. Roland Wolf, from Cancer Research UK, University of Dundee, Scotland, United Kingdom. All treatments were delivered in the presence of phenol red-free DMEM medium (Sigma).

### Microscopy

All phase contrast microscopy images were taken at either 10X or 40X magnification and images were acquired from three different fields for each sample from three independent experiments. The analysis of images was done by Image J software. Confocal microscopy images were obtained as described previously^49^.

### Western blotting

Cell lysates were collected in RIPA buffer (150 mM NaCl, 1.0% NP-40, 0.5% sodium deoxycholate, 0.1% SDS (sodium dodecyl sulphate)and Western blotting was performed as previously described^49^.

### MTT assay for cell viability

10,000 cells were seeded into each well of a 96 well plate in the presence of 100 μl medium without serum. Cells were allowed to adhere to the well overnight after which the wells were washed and treated accordingly. At the end of the treatment the media were removed and replaced with 1 mg/ml MTT (prepared in the same medium) and incubated for 2 h. Finally the MTT containing medium was removed and the purple formazan formed inside cells was extracted with DMSO and its absorbance was measured at 570 nm.

### Cell proliferation assay

Cells were grown over night in a 6 well plate followed by the appropriate treatment for up to 3 days. At the end of the treatment the cells were collected by trypsinization and counted using Countess® Automated Cell Counter from Life Technologies (Gaithersburg, MD, USA) according to the manufacturer’s instruction.

### Crystal violet staining

Cells were seeded overnight in 24 well plates and treated accordingly for 24 h. The cells were then fixed with paraformaldehyde for 30 min and stained for 10 min with 0.5 mg/ml crystal violet prepared in equal volumes of methanol and water. Excess crystal violet was removed by washing three times with PBS. The crystal violet bound to cells was then extracted in 10% acetic acid and its absorbance was measured at 600 nm.

### Quantitative real-time PCR

qRT-PCR was done as previously described^49^,^77^ and results were expressed relative to housekeeping gene, cyclophilin A (CypA). Equivalent results were also obtained using hypoxanthine phosphoribosyltransferase (HPRT). Results that compare the relative expression of genes between mouse and humans cells were expressed relative to GAPDH (glyceraldehyde 3-phosphate dehydrogenase). The primers used for RTPCR were ordered from Integrated DNA Technologies (Coralville, Iowa, USA) and the primer sequences are as follows: Human & mouse ERα: sense 5′-CCTGGACAAGATCACAG-3′ antisense 5′-AGCAGGTCATAGAGGGG-3′, Human & mouse GPR30: sense 5′-CCTGTACTTCATCAACCTG-3′ antisense 5′-TCATCCAGGTGAGGAAG-3′, Human & mouse GAPDH: sense 5′-ACTTTGTCAAGCTCATTTCC-3′ antisense 5′-TCTTACTCCTTGGAGGCCAT-3′, Mouse Nrf2: sense 5′-CAAGACTTGGGCCACTTAAAAGAC-3′ antisense 5′-AGTAAGGCTTTCCATCCTCATCAC-3′, Mouse NQO1: sense 5′-AGCTGGAAGCTGCAGACCTG-3′ antisense 5′-CCTTTCAGAATGGCTGGCA-3′, Mouse HMOX1: sense 5′-GCTAGCCTGGTGCAAGATACTG-3′ antisense 5′-CACATTGGACAGAGTTCACAGC-3′, Mouse ABCC1: sense 5′-GCGCTGTCTATCGTAAGGCT-3′ antisense 5′-AGAGGGGCTGACCAGATCAT-3′,

Mouse ABCG2: sense 5′-TGGACTCAAGCACAGCGAAT-3′ antisense 5′-ATCCGCAGGGTTGTTGTAGG-3′, Mouse ABCC3: sense 5′-GGGCTCCAAGTTCTGGGAC-3′antisense 5′-CCGTCTTGAGCCTGGATAAC-3′,

Mouse CypA: sense 5′-CACCGTGTTCTTCGACATCAC -3′ antisense 5′-CCAGTGCTCAGAGCTCGA AAG -3′, Mouse SOD1: sense 5 ‘-CCA GTG CAG GAC CTC ATT TT-3′ antisense 5′-CAC CTT TGC CCA AGT CAT CT-3′, Mouse HPRT: sense 5′-GCTGGTGAAAAGGACCTCT-3′ antisense 5′-CACAGGACTAGAACACCTGC -3′.

### Measurement of ceramide concentrations by LC-MS/MS-MRM

Standards of C16:0-, C22:0-, C24:1- and C24:0- ceramides plus C16:0- and C24:0-dihydroceramides were diluted with methanol to prepare calibration solution mixtures with concentrations of 2, 1, 0.5, 0.2, 0.1, 0.05, 0.02 and 0.01 pmol/μl of each component. These were stored at −20 °C prior to use. Lipids were extracted from treated cells using a modified Bligh and Dyer extraction method using 1 ml of methanol, 1 ml of chloroform and 0.9 ml aqueous solution (2 M KCl/10 mM HCl solution). For the analysis, 800 μl of the chloroform phase was then aspirated, dried under N_2_ and then redissolved in 100 μl methanol. C17:0 ceramide was used as the internal standard at a concentration of 0.1 pmol/μl in all standard and sample solutions. *LC conditions:* Separation of ceramide species was performed on an Agilent 1200 series HPLC system (Agilent Technologies, Palo Alto, CA) using an Ascentis C18 column (5 cm × 2.1 mm I.D., 3 μm particle size, Supelco, Bellefonte, PA). The mobile phase consisted of (A) 0.1% formic acid in water and (B) 0.1% formic acid in a mixture of acetonitrile and 2-propanol (40:60, v/v). The flow rate of mobile phase was 0.3 ml/min and the injection volume was 5 μl. Chromatographic analysis was performed using the following gradient: 0–1 min, 50% B; 1–4 min, 50% to 100% B; 4–12 min 100% B. The column was then re-equilibrated at the initial conditions (50% B) for 5 min prior to the next analysis. *MS*/*MS conditions:* MS analysis was performed on a 3200 QTRAP mass spectrometer (AB SCIEX, Concord, ON, Canada) using Analyst 1.4.2 software. The mass spectrometer was operated using positive ion electrospray ionization (ESI) in the multiple reaction-monitoring (MRM) mode. Nitrogen was used as curtain gas (CUR), nebulizer gas and drying gas. The instrumental parameters were set as follows: CUR, 10 psi; collision gas (CAD), 5; ionspray voltage (IS), 5200 V; temperature (TEM), 400 °C; Gas 1, 50 psi; Gas 2, 60 psi.

### Luciferase Assay

MCF-7 cells stably expressing the ARE sequence upstream of the reporter gene were used for the luciferase assay experiments. 500,000 cells were seeded in 6 well plates and grown overnight before adding treatments. The cells were left in the treatment medium for 24 h after which they were washed twice and collected for the luciferase assay, which was performed according manufacturer’s instructions with a kit from Promega Corporation (Madison, WI, USA).

### Superoxide measurement

Cells were grown in a 12 well plate to about 50% confluence after which the media were removed and the cells were washed with HBSS (Hank’s Balanced Salt Solution) followed by a 1 h pretreatment with 25 μM dihydroethidium (DHE), from Life Technologies. Cells were then treated as described for 24 h in DMEM starvation medium. The signal intensity, which is a measure of superoxide formation, was analyzed after taking images with a live cell fluorescent microscope (Leica Microsystems, Concord ON, Canada) with 40× magnification. Images were acquired using Openlab 4.0.2 software. Signal intensity was quantified using Image J software from 3 independent experiments with images taken from 3 different fields for each treatment condition.

### Establishment of orthotopic tumors and administration of tamoxifen in mouse

Female Balb/c mice were purchased from Charles River (Kingston, ON, Canada). At 10-week of age mice were orthotopically injected in the mammary fat pad with syngenic mouse 4T1 breast cancer cells as previously described^77^. Tamoxifen was prepared in 100% peanut oil at a stock concentration of 50 mg/ml. We treated mice with a loading dose of 400 mg/kg for 2 days, followed by a maintenance dose of 200 mg/kg for 4 days and finally at 100 mg/kg for the next 4 days. Mice in the control group were gavaged with just peanut oil. After 10 days of treatment the mice were euthanized and the primary tumors were excised and weighed. The studies on mice complied with the Canadian Council of Animal Care as approved by the University of Alberta Animal Welfare Committee (Animal User Protocol 226).

### Patient gene microarray data collection

Gene expression microarray analysis was performed on tumors from 176 primary, treatment-naive breast cancer patients, obtained through the Canadian Breast Cancer Foundation Tumor Bank (Edmonton, AB, Canada) with approval from the Health Research Ethics Board of Alberta: Cancer Committee (ID 26195). Total RNA from tumors was isolated from frozen human breast tumor biopsies and mRNA levels of each gene were determined based on normalized gene microarray signal intensity as previously described^50^. Receiver Operating Characteristic (ROC) curve analysis was used to determine the cut-off point for each gene to categorize the values into “high” or “low” levels.

### Statistical analysis

All results are reported as means ± SEM from n ≥ 3. P values were determined by t-test or ANOVA for multiple comparisons. Values of p < 0.05 was considered statistically significant. GraphPad Prism software version 5.0a was used to plot the graphs and to calculate statistics. MedCalc software version 15.4 (Ostend, Belgium) was used for analyzing human patient survival data and statistics were calculated using logrank test on Kaplan-Meier survival curves.

## Additional Information

**How to cite this article**: Bekele, R. T. *et al.* Oxidative stress contributes to the tamoxifen-induced killing of breast cancer cells: implications for tamoxifen therapy and resistance. *Sci. Rep.* **6**, 21164; doi: 10.1038/srep21164 (2016).

## Supplementary Material

Supplementary Information
