# Supplementary material for: Oxidative stress contributes to the tamoxifen-induced killing of breast cancer cells: implications for tamoxifen therapy and resistance
Source: Sci Rep. 2016 Feb 17;6:21164. doi: 10.1038/srep21164 (PMC4756695; doi:10.1038/srep21164)
Supplement: Supplementary Information [file srep21164-s1.pdf]

SUPPLEMENTARY MATERIAL

Oxidative stress contributes to the tamoxifen-induced killing of breast cancer cells: implications for tamoxifen therapy and resistance

Raie T. Bekele, Ganesh Venkatraman, Rong-Zong Liu, Xiaoyun Tang, Si Mi, Matthew G. K. Benesch, John R Mackey, Roseline Godbout, Jonathan M. Curtis, Todd P.W. McMullen, David N. Brindley

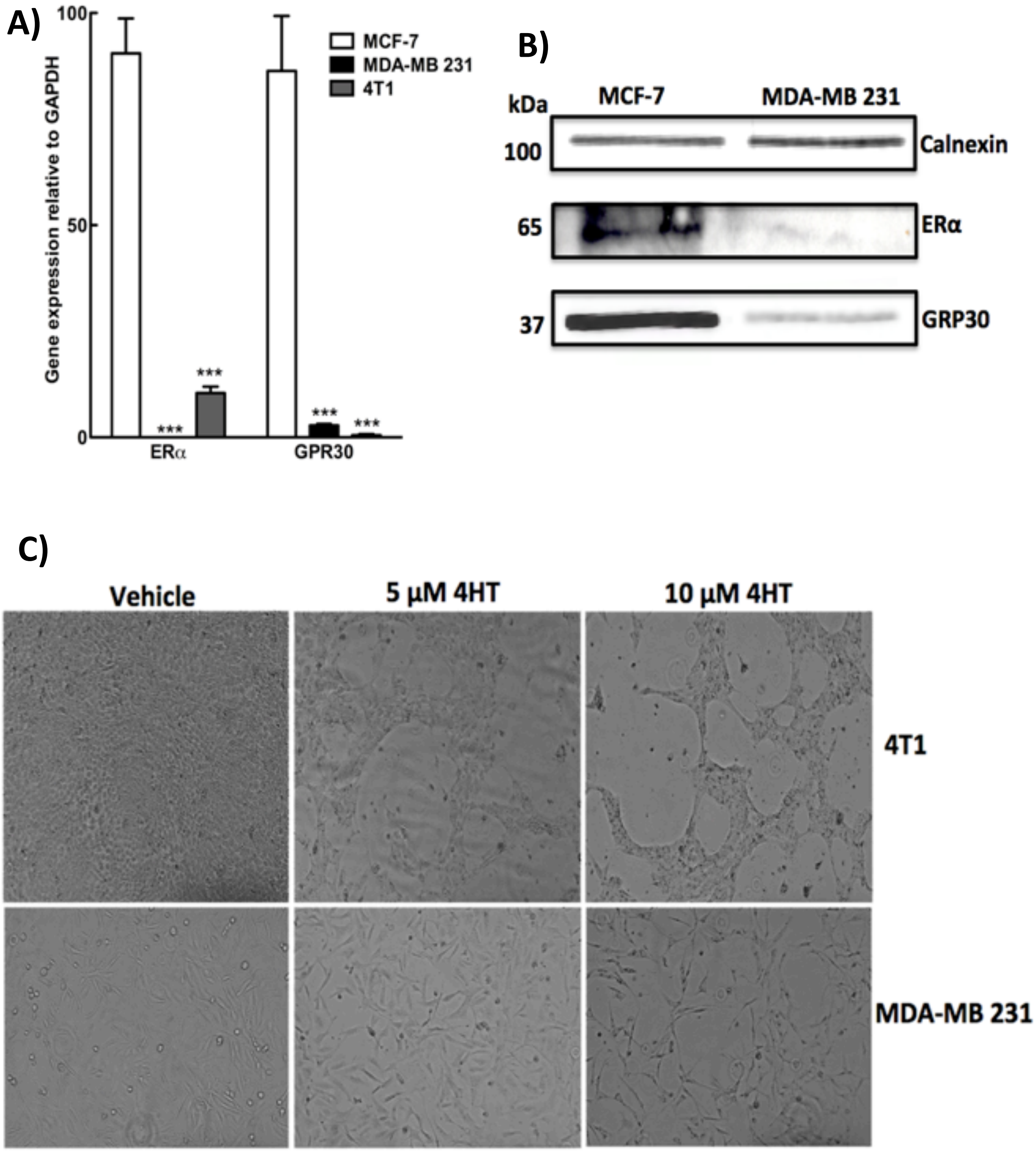

Supplemental Figure 1: Expression levels of ERα and GPR30 and effects of tamoxifen on ERα negative breast cancer cells. (A) mRNA expression levels for ERα and GPR30 in MCF-7, MDA-MB 231 and 4T1 cells. (B) ERα western blot for human breast cancer lines MCF-7 and MDA-MB 231 cells. (C) 4T1 (top) and MDA-MB 231 cells (bottom) were grown in 10% serum DMEM media for 3 days in the presence of vehicle, 5µM or 10µM of 4HT. Phase contrast microscopy images were taken with 10X objective lens and representative images from three independent experiments are shown.

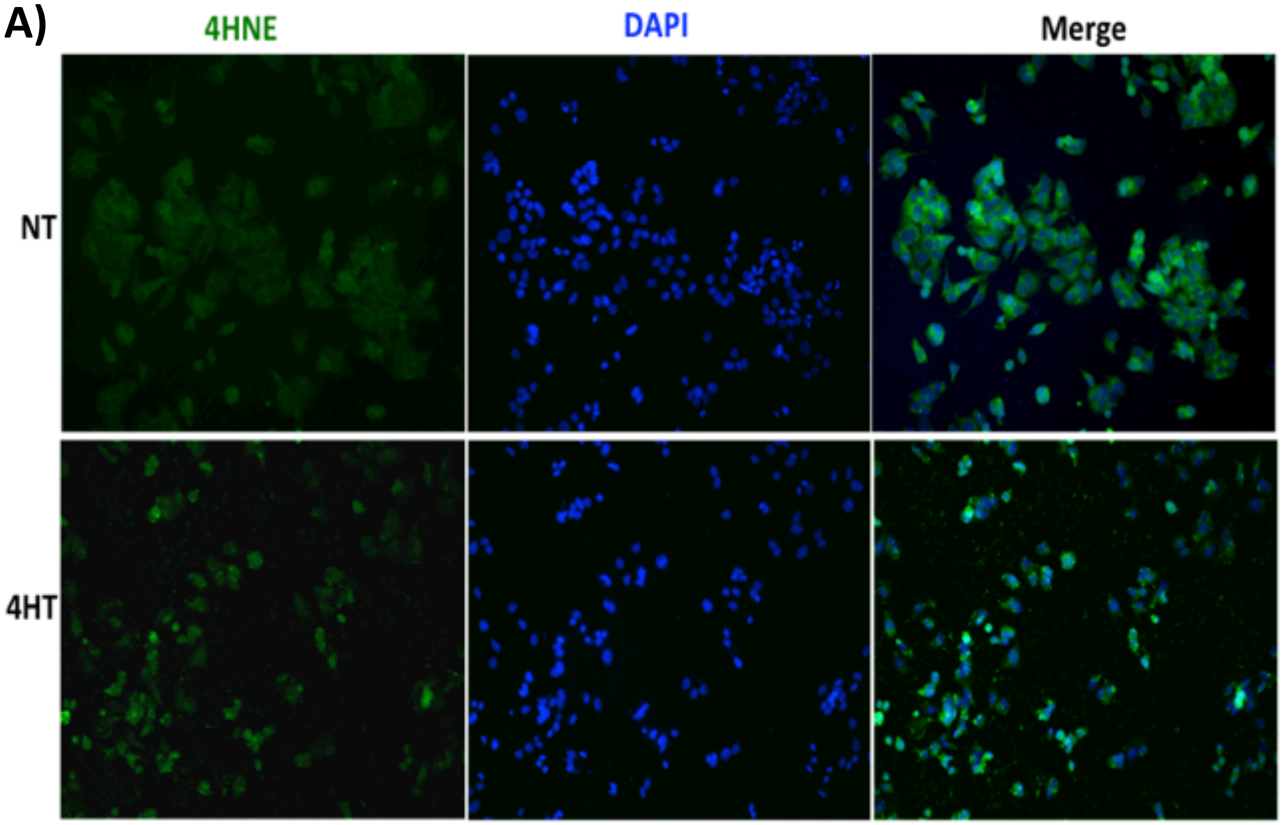

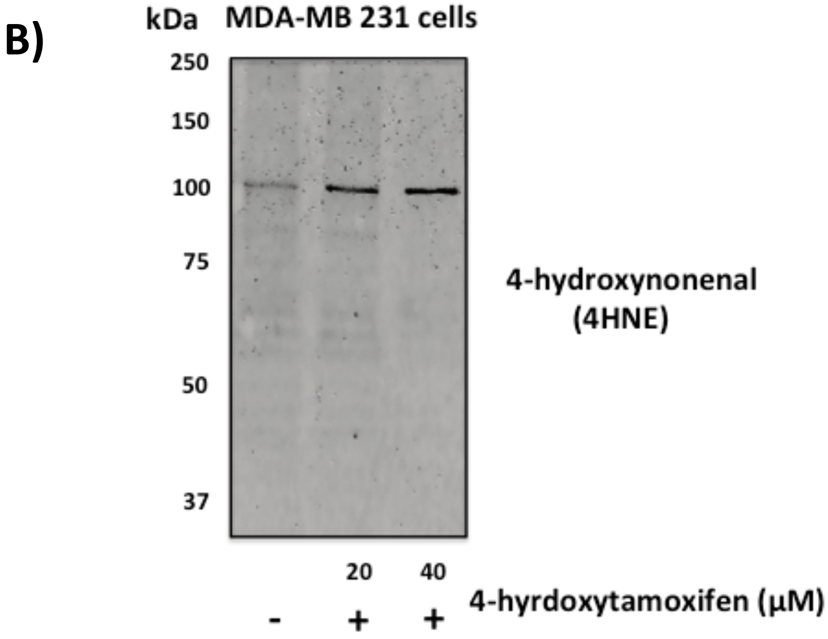

Supplemental Figure 2: Induction of 4HNE in the presence of 4-hydroxytamoxifen. (A) MCF-7 cells treated with either vehicle or with 5 µM 4HT for 24 h and cells were stained with 4HNE antibody and then counterstained with DAPI for nuclei staining. Immunofluorescence images were then acquired by staining with secondary antibody conjugated to Alexa Fluor 488 secondary antibodies. B) MDA-MB-231 cells were treated overnight with vehicle, 4HT (20 or 40µM) and followed by western blotting to measure conjugation of cellular proteins with 4HNE.

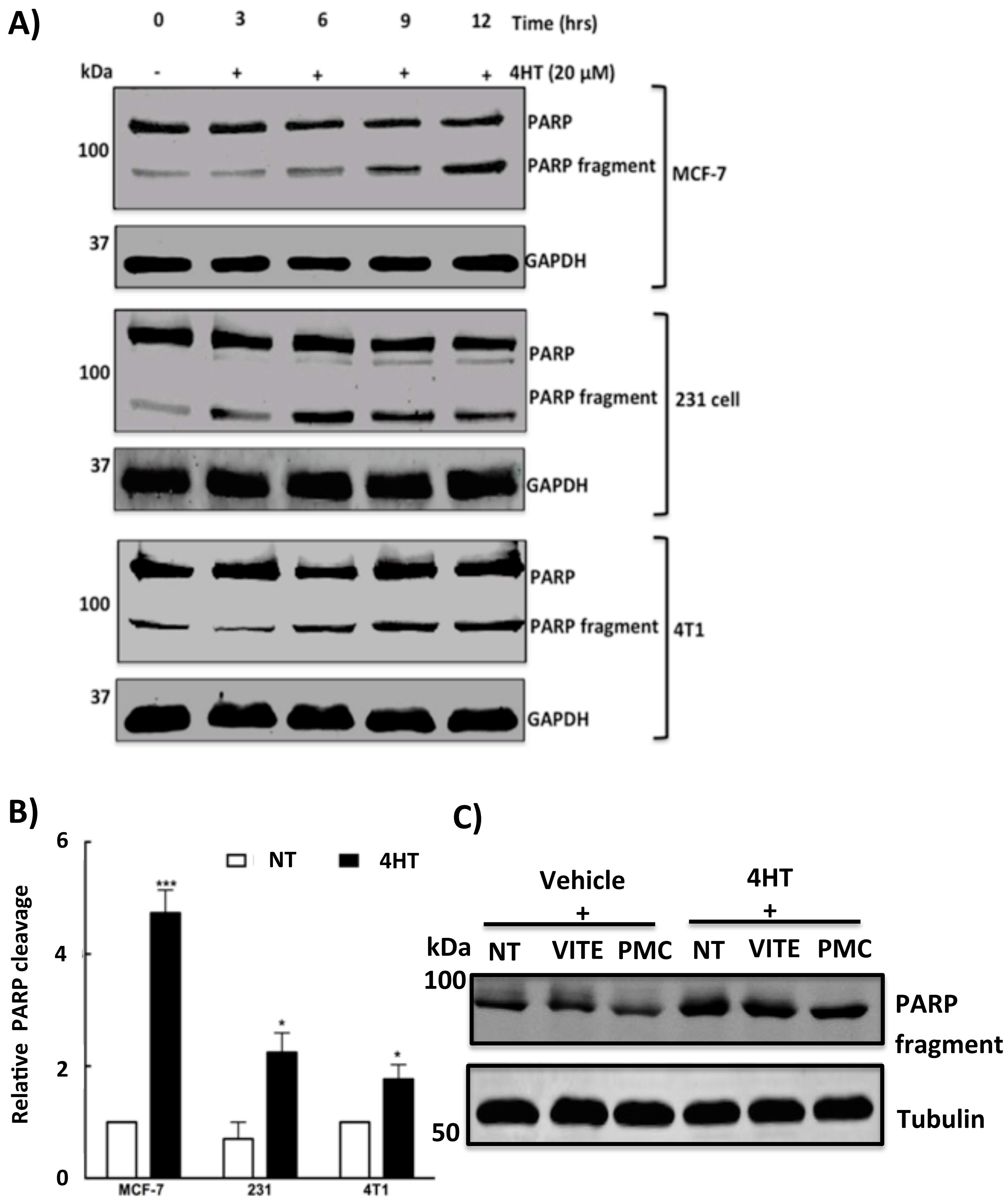

Supplemental Figure 3: **Tamoxifen increases PARP cleavage, which is not blocked by vitamin E or PMC.** (A) MCF-7 cells (top), MDA-MB-231 cells (middle) and 4T1 cells (bottom) were treated with 20 µM 4HT after which PARP cleavage was assessed by western blotting (B) Western blot quantification of the relative cleaved PARP levels was estimated from three experiments. (C) Treatment of 4T1 cells with 10 µM 4HT increased PARP cleavage but this increase is not blocked by either 100 µM vitamin E or 100 µM PMC.

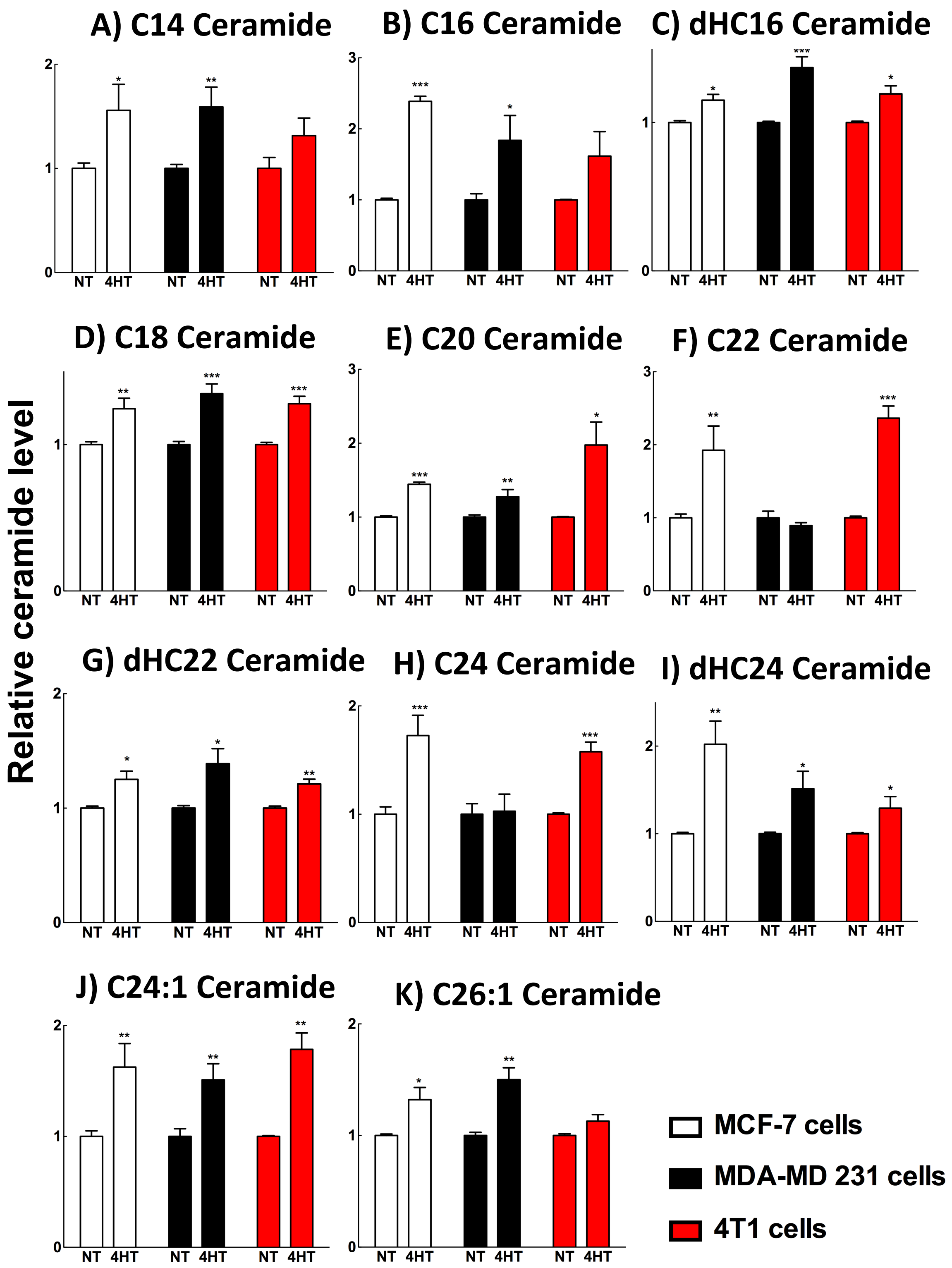

Supplemental Figure 4: **Tamoxifen increases the accumulation of different ceramide species.** MCF-7 cells indicated by white bar, MDA-MB-231 indicated by black bar and 4T1 indicated by red bar were treated for 12 h with 20 μM 4HT. Then the relative ceramide levels were assessed by mass spectroscopy for (A) C14-ceramide, (B) C16-ceramide, (C) dihydro-C16-ceramide (dHC16), (D) C18-ceramide, (E) C20-ceramide, (F) C22-ceramide, (G) dihydro-C22-ceramide (dHC22), (H) C24-ceramide, (I) dihydro-C24-ceramide (dHC24), (J) C24:1-ceramide, (K) C26:1-ceramide. All results are expressed as means ± SEM for n =4. Significant differences were indicated with * = $p<0.05$, **= $p<0.01$ and ***= $p<0.001$.

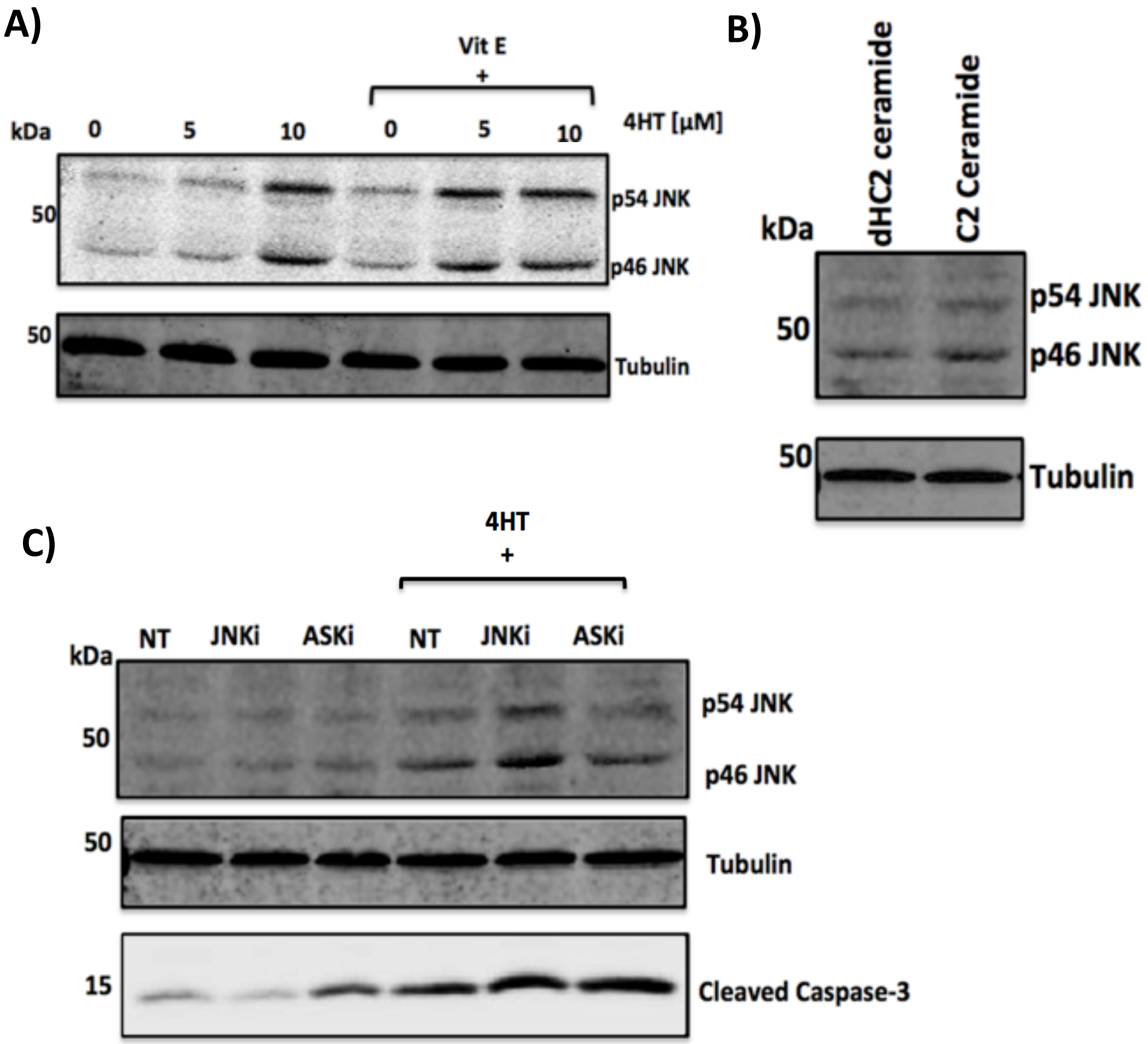

Supplemental Figure 5: **The effects of vitamin E and inhibition of ASK1 and JNK on the effects of tamoxifen.** (A) MCF-7 Cells were treated with 4HT as indicated in the presence or absence of 100 µM vitamin E and the levels of phosphorylated p54 JNK and p46 JNK were assessed with western blotting. (B) 4T1 Cells were treated with the bioactive C2-ceramide or with the inactive control dihydro-C2-ceramide (dHC2 Ceramide) followed by western blot for phosphorylated p54 JNK and p46 JNK. (C) 4T1 Cells were treated with vehicle, inhibitor for JNK (JNKi) and ASK1 (ASKi) in the presence or absence of 10 µM 4HT followed by western blot for JNK phosphorylation and cleaved caspase-3.

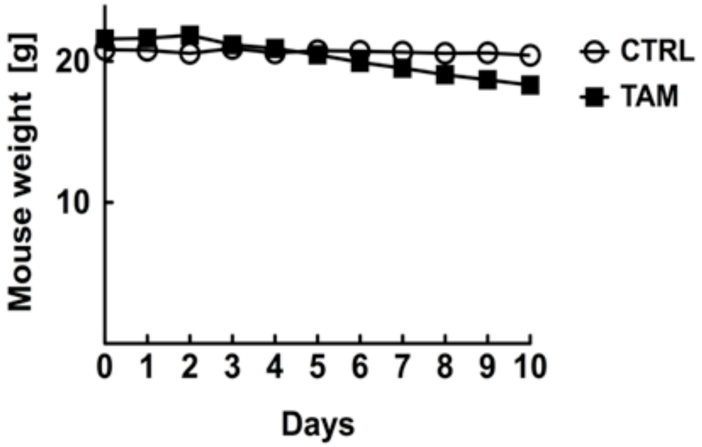

Supplemental Figure 6: Tamoxifen treatment regimen is well tolerated in Mice. To gauge the health status of mice body weight was measured every day before treatment gavage. Results were from n=6 mice in each of the control and tamoxifen treatment group.

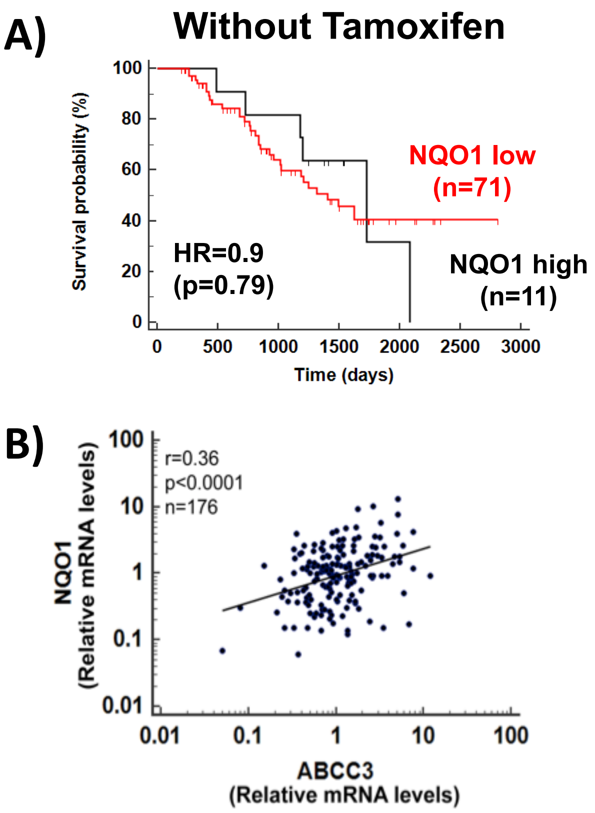

Supplemental Figure 7: NQO1 is not prognostic for treatments without tamoxifen but moderately correlates with expression profile of ABCC3. (A) Patients that are not receiving tamoxifen treatment were stratified as high and low expressers for NQO1. (B) Correlation between the expression levels of ABCC3 and NQO1 in breast cancer biopsies at original diagnosis. The correlation analysis was performed on breast tumors derived 176 patient samples.

Factors	Criterion	N	Relative %
**ER α**	Negative	64	36
	Positive	112	64
**PR**	Negative	82	47
	Positive	94	53
**HER2**	Negative	146	83
	Positive	30	17
**Age**	≤60	127	72
	>60	49	28
**Gender**	Female	176	100
	Male	0	0
**Patient status**	Alive	119	68
	Deceased	57	32
**Cancer stage**	I	75	43
	II-III	101	57
**Nuclear grade**	Low	44	25
	High	132	75
**Mitotic grade**	Low	78	44
	High	98	56
**Arch grade**	Low	29	16
	High	147	84
**Overall grade**	Low	56	32
	High	120	68

Supplemental Table 1: Clinical and pathological features of the breast cancer patients in the Breast Cancer Relapsing Early Determinants study. Breast cancer patient cases were selected for high cellularity (70% malignant cells in sample). Abbreviations for Progesterone Receptor: PR and Human Epidermal growth factor Receptor 2: HER2.
